# How U.S. children’s hospitals define population health: a qualitative, interview-based study

**DOI:** 10.1186/s12913-018-3303-7

**Published:** 2018-06-26

**Authors:** Daniel Skinner, Berkeley Franz, Matthew Taylor, Chantelle Shaw, Kelly J. Kelleher

**Affiliations:** 1 Heritage College of Osteopathic Medicine, Department of Social Medicine, Ohio University, 6775 Bobcat Way, MEB1-452, Dublin, OH 43016 USA; 2 Heritage College of Osteopathic Medicine, Department of Social Medicine, Ohio University, Grosvenor 311, Athens, OH 45701 USA; 3 Heritage College of Osteopathic Medicine, Ohio University, 6775 Bobcat Way, Dublin, OH 43016 USA; 4 Center for Innovation in Pediatric Practice, The Research Institute at Nationwide Children’s Hospital, 700 Children’s Drive, Columbus, OH 43205 USA

**Keywords:** Children’s hospitals, Hospitals, Population health, Population health management, Community benefit

## Abstract

**Background:**

The literature suggests that although adult hospitals are establishing population health programs around the country, there is considerable definitional ambiguity regarding whether interventions are aimed at the social determinants of health or the management of existing patient populations. U.S. children’s hospitals also undertake population health programs, but less is known about how they define population health. The purpose of this study is to understand how U.S. children’s hospitals define population health, and how institutions are adjusting to new preventive health care models.

**Methods:**

We conducted semi-structured interviews with key stakeholders at ten hospitals with the highest amount of staff time dedicated to population health activities as reported in the 2016 Children’s Hospital Association’s population health survey. Using a semi-structured interview guide, we interviewed representatives from each hospital. Verbatim interview notes were coded and analyzed using the data analysis software Dedoose. Data analysis followed a modified constructivist grounded theory approach.

**Results:**

Our results suggest that even population health innovators employ a variety of approaches that span both population health management and public health. We present further evidence that U.S. children’s hospitals are actively debating the definition and focus of population health.

**Conclusions:**

Definitional debates are ongoing even within children’s hospitals that are dedicating significant resources to population health. Increased clarity on the conceptual boundaries between population health and population health management could help preserve the theoretical differences between the two concepts, especially insofar as they mark two quite different long-term visions for health care. Without agreement about the meaning of population health within and among institutions, hospitals will not be able to know whether projects aimed at addressing the social determinants of health are likely to improve the health of populations.

**Electronic supplementary material:**

The online version of this article (10.1186/s12913-018-3303-7) contains supplementary material, which is available to authorized users.

## Background

In recent years hospitals have begun to engage their surrounding communities to strengthen outreach and preventive health efforts. These efforts are mostly being undertaken in the name of improving “population health,” a term that has arisen in contrast to hospitals’ traditional focus on direct patient care. As Lawrence Casalino and colleagues begin their recent article on the subject, “Everyone in health care is working to improve population health these days. Or will be very soon. Or feel that they ought to be” [1].

A widely-referenced definition of population health comes from Kindig and colleagues, who describe this concept as “the health outcomes of a group of individuals, including the distribution of such outcomes within the group” [2]. In their understanding, population health emphasizes well being and quality of life and includes activities beyond those traditionally associated with hospitals, addressing the needs of populations beyond those of a hospital’s patient population. Kindig and colleagues [2] view population health improvement as a shared responsibility of health-care, public health, and community-based organizations. This perspective has been shared more recently by Pennel and colleagues [3]. At the foundation of the population health approach is a commitment to addressing the social determinants of health and preventing illness before it develops [4]. Examples of social determinants that may be targeted by hospitals in population health approaches include housing, education, employment, and food access [5].

Although there has been a more recent focus on population health, for many decades hospitals have been involved in what is more traditionally known as “population health management” and these efforts have intensified in recent years leading to potential confusion of this approach with population health. Although these terms sound similar, they are conceptually distinct. Population health management refers to programs and services primarily focused on existing patient populations for which the hospital stands to benefit directly (financially as well as in terms of outcomes, leading to reduced emergency department admissions, for example) as their health improves [6]. This focus on patient populations stands in contrast to hospital initiatives aimed at improving health outcomes for broader populations that surround the hospital. The latter is the aim of more recent population health efforts.

One common example of population health management is the rise of hospital participation in Accountable Care Organizations (ACO) in which particular patient populations (Medicaid populations, e.g.) are the focus [1, 7]. Although ACOs existed prior to the Affordable Care Act (ACA), the ACA dedicates specific sections to the establishment of ACOs in order to intensify their development. Although the ACA included incentives for pediatric ACOs, these were never funded, thereby keeping this vision from being fully realized [8]. Nonetheless many health systems have adopted adult models and shared case studies of new preventive services both within schools and in the community [8–10].

A number of legislative developments subsequently followed suit to move hospital initiatives beyond the management model and toward population health. For example, recently-issued Internal Revenue Service (IRS) requirements mandate that all nonprofit hospitals conduct Community Health Needs Assessments (CHNAs), which provide mechanisms with which hospitals can address the broader determinants of health and undertake preventive action. Even before these federal requirements, which arose as a result of the ACA, hospitals across the United States, including children’s hospitals, had begun to identify population health as a key part of their community engagement activities and strategic planning [11]. Such programs have included hospital partnerships to build community gardens [12] improve housing stock [13], reduce crime [14], and encourage exercise [15].

The literature on adult hospitals and population health is growing. For example, scholars have published case studies of innovative preventive work being undertaken by hospitals, but have also provided preliminary evidence regarding the types of adult hospitals that are most likely to engage in population health efforts. There is recent evidence that large, non-profit hospitals located in urban areas are taking the lead in population health efforts, perhaps due to increased resources [16, 17]. Other evidence suggests that FTEs dedicated to population health and support from institutional leadership are important criteria for undertaking population health work [16]. We also know that new ACA requirements for hospitals to undertake CHNAs has not significantly increased investments in population health as opposed to patient care [18].

Despite this growing body of literature related to adult hospitals and population health, very little is known about how children’s hospitals view population health or include it as part of their federally mandated community benefit efforts. There is reason to believe that children’s hospitals would be active in population health efforts given their mission and focus on the environment during child development. Indeed, the average children’s hospital spends over $100 million a year on community benefit programs, according to the Children’s Hospital Association [19]. These programs include community support, health improvement advocacy, physical and environmental improvement, and economic development [19]. A 2017 report published by the American Hospital Association found that children’s hospitals spend a larger percentage of their total expenses on community benefit than do adult general hospitals. In this study, the community benefit expenditures averaged 13.3% of their total expenses as compared to adult general hospitals spending 12.8% and teaching hospitals spending 11.3% of their total expenditures [20]. These data, coupled with findings from the 2015 Children’s Hospital Association Population Health Survey [21], constitute evidence that children’s hospitals are engaged with improving the health of their communities. However, we still do not know how children’s hospitals frame population health and the extent to which there is support within these hospitals for engaging these new models of health care. This study aims to understand how ten children’s hospitals define population health as they adjust to this new framework.

## Methods

This project followed a modified constructivist grounded theory approach which aims to uncover how individuals construct meaning. We chose this approach because we wanted to understand how hospital employees define population health and are changing internally to align practices with this new framework. This project was carried out with logistical support from the Children’s Hospital Association, which agreed to include an additional question in their 2015 survey of population health engagement by its member hospitals (see Additional file 1: Figure S1). The question we inserted asked respondents if Ohio University faculty could contact them for a follow-up interview related to their population health practices. 67 of 73 consented to future contact for a follow-up study. Although the survey results provided quantitative data about the focus of hospitals’ population health efforts (specified geographic areas, target populations, population health metrics used, and current and future plans to staff for population health) [21], the survey did not allow for hospitals to elaborate on the meaning of population health within their organization. Such description is the focus of this project. This study was approved by the Ohio University Institutional Review Board in 2016.

### Sample

Our sample includes representatives from ten U.S. children’s hospitals identified using data from the CHA’s 2015 Population Health Survey. Because we wanted to identify hospitals that were invested in population health work, we selected those who reported the highest numbers of full time equivalent (FTE) employees dedicated to population health. We use FTEs dedicated to population health as a selection criteria for several reasons. First, and most importantly, the dedication of multiple employees to population health work is evidence of institutional commitment. Second, more than formal commitment, the existence of these employees suggests that these hospitals may be engaged in population health projects that can help us to better understand different models of hospital-led or hospital-partner collaborations.

We included the top third of hospitals who participated in the CHA membership survey in our sample to ensure diversity in both hospital size and geographic region. In total, 50 hospitals provided FTE information, with the remaining 23 selecting “don’t know” or leaving the question blank. The top 30% yielded a sample of 15 hospitals, though two did not consent to have their information shared with our study team, reducing the sample size to 13. Of the 13, ten agreed to participate in an in-depth interview with the research team. Hospitals that did not agree to participate simply did not respond to the request or stated that they did not have time to complete the follow-up interview. The hospitals that participated in the original CHA population health survey identified FTEs ranging from zero to 135, with a median of three (Table 1). To protect hospital representatives’ identities, we use number identifications instead of names in our analysis. Table 2 details the hospital and participant demographics.

**Table 1:** Number of FTEs devoted to population health as self-reported in CHA annual membership survey

# of FTEs	# of Hospitals, *N* = 73
0	12
.1–6	23
7–10	6
11–20	3
21–30	3
31–40	0
41–50	0
51–60	0
61–70	1
71–80	1
81–90	0
91–100	0
101+	1

23 hospitals either did not know the total FTEs devoted to population health or left this question blank

**Table 2:** Hospital and Participant Demographics

Region	Operating Status	Participant Type	Size
Midwest	Independent	Community and/or Population Health	Large
Midwest	Adult System	Physician	Small
Midwest	Independent	Community and/or Population Health	Medium
Midwest	Adult System	Community and/or Population Health	Medium
Midwest	Independent	Physician	Medium
West	Adult System	Community and/or Population Health	Large
West	Independent	Business/Strategic Planning	Large
South	Independent	Business/Strategic Planning	Large
South	Independent	Community and/or Population Health	Large
Northeast	Independent	Government Relations	Medium

Definitions**-** Hospital Size: 1–249 = Small, 25–424 = Medium, 425+ = Large, Operating status: Independent refers to a free-standing children’s hospital, whereas Adult System refers to children’s hospitals who are part of a larger adult hospital system

### Data collection

As the CHA’s membership survey specifically addressed population health, as defined by Kindig and others [2], hospitals were asked to identify the person most familiar with the full scope of the institution’s work in this area to complete the survey. Once consent was received by participants, a phone interview was scheduled with the same hospital employee who had completed the CHA annual membership survey. In contacting that same person, we are confident that we interviewed the person best situated to speak to the hospital’s overall programming and approach, even if that person may not be the most intimately involved in the actual day-to-day implementation of those programs. The interviews were led by the first or second author, a political scientist and medical sociologist, respectively. The interviewers asked questions related to current population health programs, staffing, and definitions of population health. See Additional file 1: Figure S2 for the complete Interview Guide. Extensive notes and verbatim quotations were taken by the first two authors, as well as two second year medical students who observed each phone interview. These students were trained in qualitative data collection and analysis, as well as research ethics. Due to advice from children’s hospital professionals regarding potential discomfort among hospital personnel, and as a condition for utilizing their membership survey, these interviews were not recorded. Interviews averaged 60 min. After each interview was completed, the combined notes were compared to ensure accuracy and consistency, and uploaded to the online data analysis software, Dedoose.

### Data analysis

The interviews were coded using procedures drawn from constructivist grounded theory analysis [22]. In this framework, simultaneous data collection and data analysis occurs. This means that interviews are coded immediately after completion and preliminary findings are used to guide additional interviews and adjust the interview guide. The constructivist variant of grounded theory emphasizes the construction of meaning, by interview participants as well as the researchers. This method was appropriate for this study because we aimed to understand how population health was conceptualized in children’s hospitals and also acknowledge our own potential biases in interpreting the findings given our background as social scientists studying medicine.

The same four researchers who participated in the interviews coded all ten sets of notes. Coding occurred in three phases, including initial line-by-line coding, secondary coding to develop categories, and final organization of codes into research themes. The first four authors met after each interview to assess the developing code book. In cases of code disagreement between researchers, meetings were held to reach consensus on coding and merge duplicative codes, which were identified using Dedoose’s code co-occurrence tool. We also utilized the inter-coder reliability function in Dedoose to ensure consistency across coders. The researchers wrote memos throughout the coding process to detail patterns in responses and ensure conceptual rigor and consistency [23]. The final product was a group of themes related to the definition of population health.

## Results

We began our study with two key assumptions. First, based on self-reported FTEs, we assumed that the hospitals had dedicated significant resources to population health initiatives. Second, due to some very large FTE numbers reported, we expected to find that these children’s hospitals were deeply involved in population health, from leadership to employees. Despite sharing a large number of FTEs devoted to population health, hospitals described disparate frameworks for understanding the role of population health in children’s hospitals. In particular, interviewees described three quite different approaches to population health: hospitals that exclusively used population health management strategies, hospitals that described their approach as population health, but are really focused on population health management, and hospitals that equate population health with public health. We have included additional participant quotations for each theme in Table 3.

**Table 3:** Additional Participant Quotations

Theme: Multiple Definitions of Population Health Operative in Children’s Hospitals	
Hospital	Quotation
CH1	“I always have to clarify that I mean population health versus population health management.”
CH2	“My concepts of population health are consistent with all children in this geographic area.”
CH4	“I think that people don’t have a solid definition of what is population health. Often times people are talking about...they may just be managing their population of asthmatics that are here…”
CH5	Population health is “a recognition that outcomes would never come without improvements to quality of life before kids enter hospital doors.”
CH6	“So many people define it differently...population health is developing a culture that will improve the overall health of a community...I don’t want to say it’s specifically just the patients...but the community. Improve the health of a community and decrease the health disparities. I would take the term ‘patients’ out of it.”
CH8	“We consider financing part of our population health function...as we grow health care plans and grow some of our own risk out there...launching a health plan for Medicaid children, the sickest of the sick...” (referring to health care plans as an example of population health).
CH9	“Taking full risk...ownership of the entire medical management of those lives...That’s the only way we’re going to truly change our outcomes.”
Theme: Support for Population Health among Hospital Administrators	
CH1	“We have not done a good job with population health measures...how do you make the case that a program is working and continue funding?”
CH3	“We’re going to need…if there is a fundamental recognition that health care is tied to socioeconomic factors…then government as a whole is going to have to drive money into those spaces more than they currently do.”
CH4	“I think it’s in an ongoing conversation...so it’s a continuum, right? We sort of, we weren’t there, but we’re making progress.”
CH5	“I think most people are coming along...there are definitely pockets of resistance. I joke about my area being an area that financial people hate...we are not the NICU revenue generator. We invest in the community, but certainly aren’t making that money back on the things we are doing in the community. That speaks to the hospital’s commitment to this..our board really integrated this into the strategic plan...really speaks to the majority of people coming along.”
CH6	Regarding their hospital mission: “Population health, rather than treating patients, is core to our foundation...I would love for us to add a fourth dimension to our [definition], but it’s falling on deaf ears…What pays off in the long run doesn’t keep the door open.”
CH7	“We’re still learning. I know there’s a lot of conversation that occurs in the executive suites when we talk about things like the social determinants of health.”
CH9	“I don’t see this ever being fully successful without that executive level commitment.”

### Multiple definitions of population health operative in Children’s hospitals

A key finding suggested the presence of considerable disagreement about the meaning of population health among the pediatric health care personnel we interviewed. Some hospitals specifically described ambiguity about and a lack of consensus over this term. As CH7 explained, “Population health means whatever you want it to, apparently.” Other hospitals described the meaning of population health as evolving within their hospital. CH6 said, “A year ago I’d give you a different answer, two years ago I’d give you a different answer, three years ago I’d give you a different answer….” Still, when pushed to provide a working definition, hospitals disagreed on whether population health referred to existing patients or the general populations of neighborhoods surrounding the hospital, or to more expansive geographies.

When describing their population health activities, some hospitals described what would most traditionally be defined as “population health management.” As CH9 explained, “if you ask 10 different people...you’re likely to get 10 different answers. Changing a hospital system of care to a full care model that extends to wherever patients might be...might mean schools, homes...really managing the patients we serve no matter where they are in the care continuum...both in terms of quality and costs.” CH8 described a similar focus on the health outcomes of existing patients, especially related to their ability to access appropriate services. They defined population health as, “Care coordination, care navigation… a lot of different buzzwords... but we consider all of those to be a part of population health.”

Other hospitals described work in public health, but described services traditionally identified with population health management. For example, CH4 described their hospital’s approach to population health as centering on their “integrated care system and so we within that system...manage over 100,000 Medicaid lives...and work with clinic and community practices.” This hospital described these largely managerial programs as concerned with “the health of the population,” akin to “more of a public health approach” than “direct clinical care.”

A third group of hospital employees explicitly distinguished their approach to population health from population health management. According to CH3, “Most hospitals when they talk about population health are actually talking about population management.” For this hospital, population health concerns “engaging in primary prevention and prevention programs in the community, with kids who hopefully never touch our hospital,” with the goal of using prevention, as “the entire aim of population health,” “to eliminate the need for hospital services.” According to an employee from CH5, population health concerns “the health of the population in our region,” which they admit “feels somewhat generic but obviously it is not just about treating disease, but it’s overall health indicators.” The employee from CH10 took a similar approach: “Many of those factors that drive health are most readily addressed at the population level, not at the individual level,” adding, “That’s why we embrace the more holistic definition of population health than the more traditional approach of condition management.”

Some of the hospitals pushing for a broader definition of population health argued that hospitals should expand their reach beyond patients and “focus on the city,” as the employee from CH1 put it. The CH5 employee similarly described population health initiatives in the “area around the hospital” as “priority one and sort of a testing ground for things we might want to think about taking to scale in the future…” Finally, the employee from CH2 pushed for a “broader sense of [population health]... looking at all the children we serve in that large geographic area…,” adding, “When people think of health care, they think about a hospital or a clinic... they don’t think about a community.” After compiling a list of hospital programs described by participants, the authors sorted these programs into categories of population health or population health management (Table 4).

**Table 4:** Examples of Hospital-led Programs across Sample

Hospital-led Program	Population Health	Population Health Management	Rationale for Categorization:
Community health workers visit repeat ER users		X	Changes utilization behavior of existing patient populations
Partner with schools to develop community gardens	X		Addresses social determinants of health among general population
Violence prevention	X		Addresses social determinants of health among general population
Patient advice line		X	Provides follow-up information to existing patient population
Follow-up telephone calls after birth		X	Provides follow-up information to existing patient population
Increasing contraceptive access in community	X		Improves access to health care among general population
School-based Clinics	X		Improves access to health care among general population
Improving patient data systems		X	Provides comprehensive data on outcomes and quality of care related to existing patient population
Improving affordable housing stock	X		Addresses social determinants of health among general population
Home visits for patients with positive lead exposure		X	Provides follow-up information/care to existing patient population
Patient and family advisory groups		X	Provides follow-up information to existing patient population
Developing ACOs: Risk-bearing and Population management models		X	Changes funding models for existing patient population
Supporting dental clinics in underserved areas	X		Improves access to health care among general population
Drowning prevention	X		Promotes accident prevention among general population
Developing walking trails for children	X		Addresses social determinants of health among general population
Referral services for patients		X	Provides follow-up information/care to existing patient population
Providing pre-school to at-risk youth	X		Addresses social determinants of health among general population

### Support for population health among hospital administrators

Regardless of how participants interpreted population health, another recurrent theme suggests that building infrastructure for population health is a challenge in children’s hospitals. Although interviewees detailed various stages along the way in transitioning to population health work, a lack of consensus regarding the priority of this work was common. In some cases, evidence suggested that defining population health in terms of improving patient outcomes and preventing readmission was more easily integrated into hospital’s strategic plans.

For example, CH10 documented a “change from a hospital-centric approach to a patient-centered, community-centric approach, where our care is really based on managing an entire population regardless of kind of where they are.” For this hospital, which identified a large number of FTEs dedicated to population health work, “The majority of [those employees] are through those integrated services.” This transition required getting out of the traditional “hospital-based thinking” that population health frameworks challenge.

Similar hospitals whose work focused more particularly on patient outcomes described transitioning to population health management as less problematic than other approaches. For example, the participant from CH4 pointed to recent expansion projects related to population health, and new employees with titles specifically referencing population health who “work with clinic and community practices.” But moving to include non-patient populations and social determinants of health is more difficult and “an ongoing conversation.” This employee explained that while hospitals are a “key community player and economic driver for the community that they serve,” it is “hard to get involved in housing when you have children dying of cancer.”

Those hospital employees who defined population health more broadly shared additional challenges in redefining their hospital’s way of thinking. The first common challenge identified was convincing hospital administrators to focus on the social determinants of health and taking on additional risk in financing initiatives beyond direct patient care. CH5, for example, has taken on neighborhood-level economic development, and uses the surrounding neighborhood as a way to pilot interventions aimed at the social determinants of health. Unsurprisingly, within these population health contexts, there is often a pull back to traditional services since, as this interviewee noted, health care is “what we know best.” The CH2 participant added that population health interventions “require broad internal buy-in as well...Eventually we’ll get there,” but “people get frightened about what we’re trying to do” and ask “why are we paying for community health workers?”

A common worry concerned investing in population health if evidence was still limited about the efficiency and sustainability of such initiatives. Some participants expressed disagreement and frustration within their institutions about whether to take the leap from population health management to broader population health strategies. As an employee from CH6 explained, “They’re defining population in a business manner and only looking at the patients in front of them...in other words, we’re only going to treat patients with population health programs for which we have a business reason to do it. That really rubs me the wrong way. It’s because of the business...the money...we’re not going to build infrastructure when we have no reason to do it from a business standpoint...if you’re long sighted and care about the community…They’re all your patients eventually.”

Similarly, the participant from CH1 described challenges posed by population health, noting, “it’s much broader...we have limited funds…without immediate outcomes...it’s much more challenging to get more funds to support that” and “to decide how much risk to take on.” Internally, the goal is to “help try and shift people to become comfortable with risk.” Whereas population health management efforts, such as those concerning ACOs, are primarily focused on assuming various levels of financial risk within defined patient populations, the risk associated with population health concerns responsibility for outcomes in communities. These activities are often tied to tax benefits for non-profit hospitals and established through federally-mandated community health needs assessments. Unlike population health management initiatives, which involve financial strategies that promise savings, even if only in the long term, population health projects are generally considered to operate at a loss initially. They are, therefore, a somewhat more direct example of community benefit.

In addition to challenges, some hospitals shared success stories in establishing population health commitments. For example, an employee from CH7 explained that “our board prioritizes re-investing resources into their community...The real role of the children’s hospital in the community is to expand the capacity of the community.” The goal is “investing in the community to make it healthiest place to raise a child.” CH3 reported that the various stakeholders within their hospital are in agreement about the future role of the hospital in population health, stating that “population health is a key strategy for entire institution, and it goes all the way up to the board.” Among other things, this means that the hospital “board itself is engaged in the approval of needs assessments and plans tied to needs assessment.” Finally, a representative from CH10 explained that when they started laying the groundwork,

“The concept of what drives health outcomes was brand new. That was daunting, unnerving to colleagues. Helping the board understand why we need to approach this differently took time, but they were and remain committed to it. The natural expectation of a traditional hospital board is by what quarter should we expect these outcomes...these outcomes happen over generations, not quarters of a year. They are there now and I’m very fortunate to have this engaged and supportive board and research team.”

Some hospitals, in other words, had committed to engaging population health projects despite facing early challenges and internal disagreement.

## Discussion

Our findings demonstrate a lack of consistency in defining population health within and between children’s hospitals. This finding mirrors similar findings in the literature on adult hospitals, with some hospitals emphasizing direct work with patient populations and others focusing on strengthening connections with public health organizations to improve community-level outcomes [24, 25]. Our study adds to this literature and demonstrates that these definitional debates are ongoing even within institutions that are dedicating significant resources to population health. These findings are important because many institutions may be using the language of population health without truly engaging upstream social determinants or expanding efforts to include the general population as opposed to existing patients.

These interviews show that some children’s hospitals view population health as an opportunity to build innovative programs to address the social determinants of health and recognize the need to change existing institutional culture to accomplish these goals. Indeed, in many cases there is a great deal of excitement about the potential role that hospitals can play in boosting health outcomes, not only among patients, but within broader communities. Other participants were in the midst of an often slow process of rethinking the role of children’s hospitals in improving health outcomes on the population, as opposed to the patient level. As our interviews show, a complicating factor in this regard was a lack of clarity about the meaning of population health itself, and difficulty in distinguishing it from the more traditional, largely business-related concept of population health management. These findings suggest that definitional ambiguity may be a critical factor in recent studies showing that U.S. hospitals have been slow to increase investments in population health and upstream social determinants despite calls to do so [16, 26]. The move to population health will likely require more pointed definitional work around population health and the relative contribution of social determinants to health outcomes, as well as an increasing commitment to FTEs dedicated to community engagement and evaluation beyond positions established in the area of population health management.

There are perceived challenges to transitioning to models that emphasize the social determinants of health, including the difficulty of measuring impact and the fact that hospitals would assume financial risk for population health improvement. Our participants identified these challenges as part of an ongoing dialogue within their institutions regarding whether to embrace a true population-based approach to health care. These findings add to the literature on hospitals and population health by providing an in-depth institutional perspective on the process of adjusting to new health care models. Despite these challenges, we argue that there is much at stake for children’s hospitals in their definitions and approaches to population health.

For example, while population health management is an important part of contemporary system-level approaches to patient populations, a critique of population health management becomes increasingly important if it eclipses or occurs at the expense of population health. Specifically, population health management strategies may be bound to narratives of individual behaviors that drive patient outcomes, as opposed to population health strategies that cast social problems (including food systems, neighborhood design and safety, and air quality) as the upstream causes of illness [27]. In population health management, efforts may focus on individual behaviors such as diet, exercise, medication compliance, or care navigation. Potentially lost in this “neoliberal” process of casting social problems as matters of individual behavior and responsibility, and turning to markets to solve them [28], is a richer understanding of how neighborhoods become havens of poverty, the causes of chronic unemployment, why a particular neighborhood becomes a food desert, or the degree to which a city’s mass transportation system may compound problems that ultimately impact health. While population health strategies may positively impact the health of individuals, the individual is not the primary focus of such engagements as they often are within population health management paradigms. Accordingly, the concept of management itself can be understood as anathema to population health when it comes to replace the broader aims of population health interventions. At their best, population health management efforts should be grasped as complementary to, and not a replacement for population health programming that promotes wellness through empowerment and the shoring up of social services. A key difference between population health management and population health lies in whether problems - including non-medical problems - that affect entire communities are considered important and are increasingly engaged by traditional medical institutions such as children’s hospitals.

Our findings indicate that while children’s hospitals with the highest number of FTEs devoted to population health are beginning to undertake work in the areas of housing, crime, community health promotion, and collaborate with important community stakeholders, there is also wide variation in how they define population health. The tendency to conflate population health with population health management could result in losing some of the important theoretical differences between the two models. Beginning with a discussion about the differences in these two theoretical paradigms would better allow children’s hospitals to participate in rethinking these institutions’ roles in public health. Although the science of population health is still developing, there is compelling evidence that social determinants account for a relatively larger portion of health outcomes than clinical care [29]. Children’s hospitals, specifically, may wish to consider the unique role that the social environment plays in child health and development and modify adult population health models accordingly. Critics have argued, for example, that population health has to date fallen short by focusing more on the measurement of existing health outcomes, rather than developing new community-level interventions to improve future outcomes [30]. Others have argued that new investments should push health care systems toward population health models, rather than just reforming existing clinical services [25, 31]. Clarifying the definition of population health within children’s hospitals will be important for the future development of programs and strategic planning.

Definitional ambiguity also poses challenges for studying the growth of population health activities among children’s hospitals. Disagreement over the meaning of population health within and among institutions, will make it difficult for researchers to grasp whether projects aimed at addressing the social determinants of health, or improving health care financing, are increasing and are effective at improving the health of populations. These findings raise additional questions such as what types of workers should count as population health staff. These questions make it difficult to assess the current state of population health in children’s hospitals and have serious implications for the evaluation of existing programs and providing comparisons between different types of institutions. For these reasons, we argue that definitional clarification will not only improve our understanding of the current state of population health efforts within children’s hospitals, but also encourage a renewed consideration of how hospitals might engage the social determinants of health to prevent illness and improve health outcomes for larger populations.

### Limitations

A study such as ours necessarily has limitations. Most importantly, our in-depth interviews were limited to ten children’s hospitals. To some extent, even within these ten, outcomes will vary depending on the particular personnel interviewed. In the case of our interviews, specific interviewees were determined by who, at each hospital, was tasked with completing the CHA population health survey. The personnel we interviewed tended, as we should expect, to be sympathetic to population health as a concept. While our study captures confusion about population health and population health management, we are not able to say with certainty, from our interviews, how pervasive this confusion may be within these institutions.

## Conclusion

We believe that these findings are important, for a number of reasons. Most importantly, to confuse the conceptual boundaries of population health and population health management is to both lose the broader approach to health that population health takes, and to undo recent efforts to expand children’s hospitals’ understanding of the roles they could play in communities and to what end. Under population health models, this requires concern for the social determinants of health in addition to direct patient care. We should expect, therefore, that children’s hospitals engaged in extensive, genuine population health work should be involved simultaneously in a radical rethinking of the very idea of what it means to be a children’s hospital in an age of population health. Only some of the hospitals interviewed were engaged in such a project, a few occupied something of a middle position, and a few simply used the language of population health while consistently describing population health management. Most hospitals interviewed described the transition to population health as slow and contentious due to the challenge this framework poses to traditional health care systems and financing--precisely where the default to population health management becomes attractive as a more familiar option.

As we have noted, these findings were striking precisely because our interviewees were selected on the basis of a self-declared commitment to population health. We believe that these and other children’s hospitals could use our findings as an opportunity to rethink their population health initiatives as well as increase communication and collaboration with other children’s hospitals engaged in expanding population health activities. At a minimum, becoming clearer on the conceptual boundaries between population health and population health management could help preserve the theoretical differences between the two concepts, especially insofar as they mark two quite different long-term visions for health care.

## Additional file

Additional file 1: **Figure S1.** Additional Question from CHA Membership Survey. **Figure S2.** Interview Guide. (DOCX 8 kb)

## Abbreviations

ACO: Accountable Care Organization
FTE: Full Time Equivalent
IRS: Internal Revenue Service
