# Supplementary material for: How U.S. children’s hospitals define population health: a qualitative, interview-based study
Source: BMC Health Serv Res. 2018 Jun 26;18:494. doi: 10.1186/s12913-018-3303-7 (PMC6019316; doi:10.1186/s12913-018-3303-7)
Supplement: Supplementary file 1 — Figure S1. Additional Question from CHA Membership Survey. Figure S2. Interview Guide. (DOCX 8 kb) [file 12913_2018_3303_MOESM1_ESM.docx]

**Figure S1: Additional Question from CHA Membership Survey**

**Study Partnership**

We have partnered with researchers at Nationwide Children’s Hospital and Ohio University’s Heritage College of Osteopathic Medicine who are conducting a study of children’s hospitals and community engagement and development. Select data from this survey will be used to identify hospitals that may subsequently be invited to participate in their study.

Please indicate your preference regarding attributable data below.

- I allow for attributable data to be shared with the researchers as described above. Data will only be used for the purposes of their study and will not be shared publicly.
- I do not want attributable data to be shared with the researchers described above.

**Figure S2: Interview Guide**

a. Population Health

1. How do you define population health?
2. Why is population health important to your hospital’s mission?
3. We identified you based on FTEs, how are employees in your hospital distributed between different departments? What are their roles? Do you have examples of what they do on a day to day basis?
4. What are some of the issues that your population health teams are addressing in their work? Can you describe a specific project?

b. Identifying Problems and Evaluating Interventions

1. What role does your CHNA process play in your population health work?
2. Does population health extend to non-medical issues?
3. What challenges exist in improving the health of populations?

c. Community Involvement

1. In developing programs do you plan to involve the community and in what way?In identifying problems? Strategies?
2. Who are some of the major community partners you work with?
3. What population do you serve?

d. Additional Questions:

1. Are various stakeholders within the hospital in agreement about the future role of the hospital in population health?
2. What types of community problems can hospitals be most effective at addressing?
3. What type of support from employees, local government, or community organizations would be helpful in developing and carrying out initiatives?
4. Have population health activities changed as a result of the ACA?
5. How do you measure improvements in population health outcomes?
6. How does funding work? Is population health work written into your strategic plan or do you have request funding for individual projects?
